# Comparative Genomics and Functional Profiling Reveal Lineage-Specific Metabolic Adaptations in Globally Emerging Fluoroquinolone-Resistant *Salmonella* Kentucky ST198

**DOI:** 10.3390/genes16091051

**Published:** 2025-09-08

**Authors:** Juned Ahmed, Rachel C. Soltys, Smriti Shringi, Jean Guard, Bradd J. Haley, Devendra H. Shah

**Affiliations:** 1 School of Veterinary Medicine, Texas Tech University, Amarillo, TX 79106, USA; junahmed@ttu.edu (J.A.);; 2 Animal Disease Diagnostic Laboratory, Department of Comparative Pathobiology, College of Veterinary Medicine, Purdue University, West Lafayette, IN 47907, USA; 3 United States National Poultry Research Center, Agriculture Research Service, United States Department of Agriculture, Athens, GA 30605, USA; jean.guard@yahoo.com; 4 Environmental Microbial and Food Safety Laboratory, Beltsville Agricultural Research Center, Agricultural Research Service, United States Department of Agriculture, Beltsville, MD 20705, USA; bradd.haley@usda.gov

**Keywords:** *Salmonella* Kentucky, ST152, ST198, metabolism

## Abstract

Background: *Salmonella* Kentucky comprises two major lineages, ST152 and fluoroquinolone-resistant (Flu^R^) ST198, which have diverged genotypically and phenotypically along distinct evolutionary and epidemiological trajectories. ST198 is linked to global human disease, while ST152 is primarily animal-associated in the U.S. We hypothesized that lineage-specific metabolic adaptations contribute to their differing host associations and pathogenicity. Methods: We performed comparative metabolic profiling of ST198 (*n* = 3) and ST152 (*n* = 4) strains across 948 substrates and environmental conditions. Growth assays tested the ability of these lineages and other non-typhoidal *Salmonella* (NTS) serovars (*n* = 5) to utilize *myo*-inositol and lactulose as sole carbon sources. Comparative genomic analyses of 294 ST198, 173 ST152, and 1300 other NTS serovars identified nutrient utilization genes. Results: ST198 exhibited significantly higher respiratory activity and broader metabolic versatility across carbon, nitrogen/sulfur sources, and stress conditions. The canonical *iol* gene cluster for *myo*-inositol catabolism was conserved in ST198 but absent in ST152, which nonetheless showed weak growth on *myo*-inositol, suggesting an alternative metabolic pathway for *myo*-inositol may exist. We also report for the first time that, despite lineage-specific differences in metabolic efficiency, multiple NTS serovars, including *S.* Kentucky, can metabolize lactulose, a synthetic disaccharide traditionally associated with beneficial gut microbes. These results suggest the potential existence of a novel lactulose metabolic pathway in NTS. Conclusions: These findings highlight ST198’s metabolic adaptability and reveal novel metabolic capacities in NTS. A mechanistic understanding of nutrient utilization pathways, particularly of *myo*-inositol and lactulose, will provide novel insights into the mechanisms underlying nutrient metabolism that likely modulate the ecological success and pathogenic potential of NTS in human and animal hosts.

## 1. Introduction

Non-typhoidal *Salmonella* (NTS), a genetically and antigenically diverse enteric pathogen with >1500 serotypes, is a leading cause of foodborne enteric illness and a significant contributor to antimicrobial resistance (AMR) worldwide, responsible for ~150 million infections and 60,000 deaths each year [1]. Among the various NTS serovars, *Salmonella* Kentucky (*S.* Kentucky) is frequently isolated from food animals in the United States, particularly chicken, turkey, swine, and cattle [2,3,4]. Two predominant genetic lineages of *S.* Kentucky, ST198 and ST152, exhibit distinct epidemiological and genetic characteristics [3,4,5,6,7,8,9]. ST152 is frequently isolated from U.S. food animals but is rarely associated with human illness. ST198 comprises two sub-clades: fluoroquinolone-susceptible (Flu^S^) ST198.1, primarily isolated from U.S. food animals [2,4,10], and fluoroquinolone-resistant (Flu^R^) ST198.2, predominantly isolated from food animals in North Africa, the Middle East, Europe, and South Asia, and often identified as a cause of human illnesses, including U.S. travelers returning from international travel [2,4,5,8,9,11].

The Flu^R^ ST198.2 sub-clade (hereafter referred to as Flu^R^ ST198) has been increasingly detected in human clinical cases globally, posing a significant public health threat [7,12,13,14]. In North America, Flu^R^ ST198 is commonly associated with travel-associated illnesses, particularly among travelers returning from North Africa, the Middle East, and South Asia, and it is often linked to the consumption of poultry or poultry-related products [2,4,9]. Notably, Flu^R^ ST198 harbors two mutations in *gyrA* and three mutations in *parC* in the quinolone resistance-determining region (QRDR), which is concerning given that ciprofloxacin remains the first-line antibiotic for the treatment of NTS infections in humans worldwide [6,15]. Recently, Flu^R^ ST198 of *S.* Kentucky was implicated in a nosocomial infection in a hospitalized American black bear at a veterinary teaching hospital in New York [16], highlighting the potential risk this lineage poses to animal health, food safety, and public health in the USA.

We previously reported that Flu^R^ ST198 has undergone microevolution driven by the accumulation of lineage-specific mutations [2,9]. Similar microevolutionary changes in other *Salmonella* serotypes and multidrug-resistant (MDR) lineages of NTS serovars, such as *S.* Typhimurium ST213/ST313 and *S.* Enteritidis ST11, exhibit enhanced metabolic capacities, improved host adaptation, and pathogenicity [17,18,19]. However, whether lineage-specific mutations among ST152 and Flu^R^ ST198 have resulted in distinct metabolic capacities remains unknown. Understanding these differences could provide critical insights into the mechanisms driving emergence, pathogenicity, and niche-specific adaptation of Flu^R^ ST198.

In this study, we hypothesized that the globally emerging Flu^R^ ST198 and domestically prevalent ST152 lineages have genetically evolved to exhibit distinct metabolic capabilities. To test this hypothesis, we conducted metabolic fingerprinting of seven epidemiologically distinct strains of ST152 (*n* = 4) and Flu^R^ ST198 (*n* = 3) by measuring respiratory activity (RA) across 948 substrates and environmental conditions. Our results demonstrate that Flu^R^ ST198 consistently exhibits significantly higher (*p* < 0.01) RA than ST152 when utilizing several carbon and nitrogen/sulfur compounds as sole sources of energy. Notably, Flu^R^ ST198 showed markedly increased RA and growth in media supplemented with two gut-associated nutrients, lactulose and *myo*-inositol, commonly used as dietary supplements. We report that the *myo*-inositol-mediated metabolic fitness of Flu^R^ ST198 and several other NTS serovars is likely due to the conserved carriage of the *myo*-inositol catabolism gene cluster (*iolCatGC*), which is completely absent in the ST152 lineage and a few other NTS serovars. Furthermore, we report that different NTS, including Flu^R^ ST198, can efficiently metabolize lactulose as a sole energy source for growth in a serotype-independent manner, due to an unknown genetic mechanism. Together, these findings suggest that Flu^R^ ST198 has acquired distinct metabolic traits that may modulate its fitness, pathogenic potential, and capacity for global dissemination.

## 2. Materials and Methods

### 2.1. Strain Selection and Growth

A subset of seven well-characterized epidemiologically and genetically distinct strains of *S.* Kentucky ST152 (*n* = 4) and ST198 (*n* = 3) were used in this study [9]. All ST198 strains in this study were resistant to fluoroquinolones (Flu^R^), exhibited multidrug resistance, and were isolated from individuals with travel histories to Asia, Africa, or the Middle East (Table 1). Conversely, all ST152 strains were fluoroquinolone-sensitive (Flu^S^), with three strains isolated from poultry sources and one isolated from a human case without travel history outside the USA (Table 1). Five additional NTS strains representing clinically relevant serovars (*S.* Typhimurium, *S.* Hadar, *S.* Enteritidis, *S.* Montevideo, and *S.* Schwarzengrund) were also tested in individual growth kinetics experiments in this study [20]. All strains were revived from −80 °C storage by streaking on tryptic soy agar (TSA) followed by incubation at 37 °C for 16 h. Colonies grown on TSA were used for Phenotype Microarray and/or growth assays.

### 2.2. Comparative Metabolic Profiling of ST152 and Flu^R^ ST198

We performed comparative metabolic profiling of ST152 (*n* = 4) and Flu^R^ ST198 (*n* = 3) strains across 948 metabolic substrates and environmental conditions using Phenotype Microarray (PM) (Biolog, Hayward, CA, USA). The PM analysis included eight metabolic panels for carbon (PM-1-2), nitrogen (PM-3, PM-6-8), sulfur (PM-4), phosphorus (PM-4), and nutrient supplements (PM-5) as energy sources. Additionally, two sensitivity panels (PM-9 and PM-10) evaluated the sensitivity of strains to osmolytes, toxicities, and various pH conditions. Testing was performed following previously established protocols [21,22,23]. Each strain was inoculated onto PM-1 to PM-10 plates and incubated at 42 °C for 48 h. Respiratory activity (RA) was measured by the reduction in tetrazolium violet dye, with color change indicating cellular respiration and electron transport chain function. The data from PM tests were organized in Microsoft Excel (Table S1) and analyzed via hierarchical clustering (Euclidean distance, complete linkage) for each nutrient/condition using RStudio (R version 4.4.1). The RA differences between ST152 and Flu^R^ ST198 were compared using Student’s *t-*test, and a mean RA difference ≥ 50 units between ST152 and Flu^R^ ST198 with *p* < 0.01 was considered statistically significant [21,22,23]. The heatmaps for hierarchical clustering were generated using the pheatmap package within RStudio (version 4.4.1) [24].

### 2.3. Benchtop Growth Assay

To confirm the differential metabolic fitness traits between ST152 and Flu^R^ ST198 strains detected by the PM array, each strain was tested in a benchtop growth assay for its ability to utilize two representative carbon compounds, including *myo*-inositol and lactulose, at mammalian (37 °C) and avian (42 °C) physiological temperatures. Briefly, bacterial colonies were revived by streaking on TSA and incubated overnight at 37 °C. Next, colonies were resuspended in M9 broth (BD, Sparks, MD, USA) to a 0.5 McFarland standard, followed by serial 10-fold dilutions to achieve approximately 200 colony-forming units (CFU)/mL. Approximately 360 CFU of each strain was inoculated into 1.8 mL of M9 media (200 CFU/mL) in 96-well blocks supplemented with varying concentrations of lactulose (1, 10, and 20 mM) and *myo*-inositol (1, 10, and 20 mM) and incubated at 42 °C for 96 h. At 24 h intervals, 100 µL from each culture was withdrawn, followed by plating of 10-fold dilutions on TSA and incubation for 16 h at 37 °C to determine the CFUs at each time point. In each of the experiments, M9 broth supplemented with glucose (1, 10, and 20 mM) was used as a positive control, M9 broth without any nutrient supplementation was used as a negative control, and glucose (20 mM) without bacterial inoculation was used as a sterility control. To determine the growth of ST152 and Flu^R^ ST198 at mammalian physiological temperature (37 °C), a single concentration of lactulose (20 mM) or *myo*-inositol (20 mM) was used, and the CFU determinations were conducted up to 72 h. Growth in each metabolite and each concentration at both temperatures was tested in duplicates in three independent experiments. Growth assay results were expressed as log_10_ CFU/mL. The growth data were organized and analyzed using Microsoft Excel (Version 2408) and RStudio (R version 4.4.1). A log_10_ difference was calculated as the difference in the log_10_ CFU between the end-time-point (72 or 96 h) and the starting inoculum (0 h). Doubling times (Td) were calculated as previously described [22] using the formula *t*/(3.3 × log(*b*/*B*)), where *t* is the end time-point for Td calculation (96 h for 42 °C growth data and 72 h for 37 °C growth data), *b* is CFU at the endpoint (72 h or 96 h), and *B* is the initial inoculum (CFU at 0 h). Log_10_ and Td differences were calculated and compared using Student’s *t*-test. A *p*-value of <0.05 was considered statistically significant. To determine the ability of other NTS serovars to utilize *myo-*inositol and lactulose for growth, we tested five NTS strains representing the following serovars (*S.* Typhimurium, *S.* Infantis, *S.* Hadar, *S.* Enteritidis, *S.* Montevideo, and *S.* Schwarzengrund). These NTS strains were grown in M9 media supplemented with lactulose (20 mM) and *myo-*inositol (20 mM) by incubating at 37 °C for 72 h. The growth of each strain was determined by calculating the log_10_ increase at 72 h post-incubation.

### 2.4. Comparative Genomics Analysis

To identify and inventory lineage-specific genes likely associated with the differential metabolic potential of ST198 and ST152 lineages, pan-genome analysis of the 7 epidemiologically distinct strains of *S.* Kentucky ST152 (*n* = 4) and Flu^R^ ST198 (*n* = 3) used in this study was performed using PanExplorer followed by gene functional enrichment analysis [25]. Follow-up comparative genomics analysis focused on determining the genetic basis of *myo*-inositol and lactulose-mediated differential metabolic fitness of ST152 and Flu^R^ ST198. To determine the genetic basis of differential utilization of *myo*-inositol between ST152 and Flu^R^ ST198 strains, we compared the genomes of the ST152 and Flu^R^ ST198 strains used in this study to identify the *iol* gene cluster (*iolCatGC*) known to contribute to *myo*-inositol metabolism and growth of *S.* Typhimurium in the presence of *myo*-inositol as the sole energy source [26,27], using MultiGeneBlast version 1.1.13 [28]. To confirm whether differential carriage of the *iol* gene cluster was consistent across ST152 and ST198 lineages, we expanded genetic screening to the publicly available whole-genome sequences of 294 ST198 strains and 173 ST152 strains along with a few other STs, including ST314 (*n* = 14), ST2132 (*n* = 3), ST19 (*n* = 2), and ST32, ST64, ST166, ST318, ST639, and ST1679 (*n* = 1 each) (Table S2), using the compare region viewer tool of the Bacterial and Viral Bioinformatics Resource Center (BV-BRC) [29]. Finally, to determine carriage of the *iol* gene cluster among other NTS serovars, a total of 1300 complete high-quality genomes representing 106 different NTS serovars (Table S2) were evaluated using the compare region viewer tool of BV-BRC.

Lactulose metabolism is widespread among gut microbes and is typically attributed to glycoside hydrolases (GHs) that hydrolyze the β-1,4-glycosidic bond of lactulose into fermentable monosaccharides (e.g., fructose, galactose) [30,31,32,33]. However, lactulose metabolism by NTS has not been described, and its genetic basis remains unknown. Thus, to identify any gene encoding a putative GH protein likely involved in lactulose metabolism in *Salmonella,* we first searched through the genome of *S.* Kentucky strain PU131, a reference strain of Flu^R^ ST198 [15]. We then searched the candidate proteins against the CAZy database to identify putative glycoside transporters and β-galactosidases that can hydrolyze the β-1,4-glycosidic bond in lactulose and metabolize it to monosaccharides [31,32,34]. To determine the carriage of putative transporter and β-galactosidase among other *Salmonella* genomes, we screened the whole-genome sequences of 294 ST198 strains and 173 ST152 strains along with a few other STs, including ST314 (*n* = 14) and other STs (*n* = 11) of *S.* Kentucky and 1300 complete high-quality genomes, representing 106 NTS serovars (other than *S.* Kentucky) (Table S3).

### 2.5. RT-qPCR

RT-qPCR was conducted to confirm the upregulation of *myo-*inositol inducible genes (*iolT1* and *reiD*) in NTS when grown in the presence of *myo-*inositol as the sole source of energy. Total RNA was extracted from a representative strain of *S.* Kentucky ST198 (PU61), which carries the *iol* gene cluster and also exhibited higher growth in the presence of *myo*-inositol as the sole energy source. Briefly, the PU61 strain was grown overnight in TSA at 37 °C. Next, ~10^8^ CFU of this strain was resuspended in M9 media containing 20 mM of *myo*-inositol or glucose (control) and incubated at 37 °C for 6 h. After 6 h, ~10^9^ CFUs were harvested by centrifugation at 10,000 RCF for 10 min at 4 °C for RNA extraction. Total RNA was extracted using the Direct-zol RNA Miniprep Plus Kit (Zymo Research, Irvine, CA, USA) following the manufacturer’s instructions with minor modifications, which included in-tube DNase treatment using Turbo DNase (Invitrogen, Waltham, MA, USA) followed by RNA clean up using the RNA Clean & Concentrator-25 (Zymo Research). An equal concentration (~820 ng) of total RNA was used to prepare cDNA using the High-Capacity cDNA Reverse Transcription Kit (Applied Biosystems, Waltham, MA, USA) according to the manufacturer’s protocol. Finally, 2 µL of cDNA and 800 nM of pre-designed primers (Table S4) for inositol catabolic genes (*iolT1* and *reiD*) were used for qPCR using the PowerUp SYBR Master Mix (Applied Biosystems) with the following conditions: 1 cycle at 50 °C for 2 min, 1 cycle at 95°C for 2 min, 40 cycles at 95°C for 15 s, and 40 cycles at 60°C for 1 min. Cq values were obtained and compared with glucose as the reference condition. Each sample was tested in triplicate. Gene expression was analyzed using the ΔCq method, which represents the difference in Cq between the test condition (*myo*-inositol) and the control condition (glucose). Log_2_ fold change was computed as log_2_ (2^−ΔCq^), and the calculation was performed in Python (version 3.11.13) using the NumPy package. The null hypothesis tested was that the mean ΔCq = 0 (i.e., no change in expression relative to glucose). A *p*-value < 0.05 was considered statistically significant.

## 3. Results and Discussion

### 3.1. Differences in Metabolic Fitness Between ST152 and Flu^R^ ST198 Lineages

**Figure 1:**
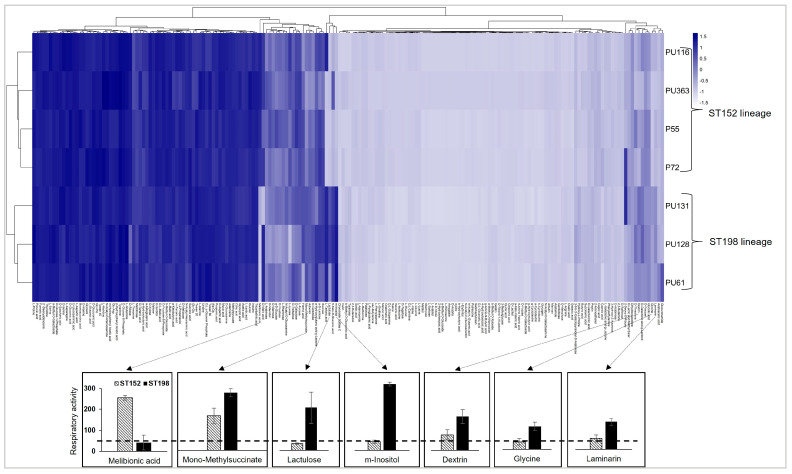
Hierarchical clustering showing distinct metabolic profiles of ST152 and Flu^R^ ST198 strains based on the differences in respiratory activity (RA) in the presence of 190 different carbon compounds as sole energy sources. The *X*-axis shows the clusters of carbon sources. The *Y*-axis shows the clusters formed by strains within each lineage tested in this study. The dark blue indicates higher RA, and the light blue to white color indicates lower RA, with the bar graphs below showing seven carbon sources that are significantly differentially utilized by ST152 and Flu^R^ ST198 strains with an RA difference of >50 at a *p*-value of ≤0.01. The *Y*-axis in each bar graph shows RA activity in the presence of each carbon source, and the dotted line represents the RA value cutoff of 50.

**Figure 2:**
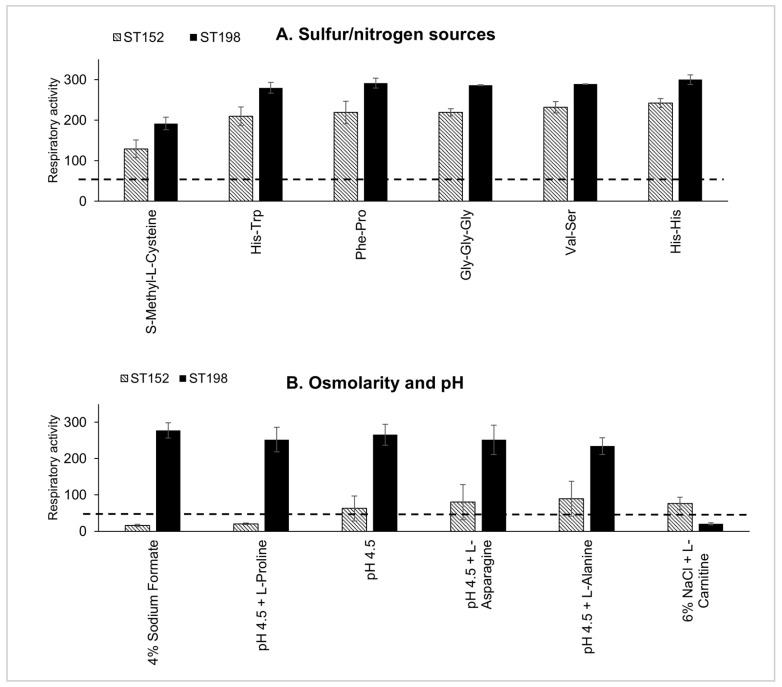
Difference in the respiratory activity (RA) between ST152 and Flu^R^ ST198 strains. ST152 and Flu^R^ ST198 strains showed significant differences (*p* < 0.01) in RA for six different nitrogen/sulfur compounds as sole energy source (**A**) and in response to six stressors (**B**). The dotted lines indicate the RA value cutoff of 50.

The metabolic fitness differences between ST152 and Flu^R^ ST198 lineages were evaluated by measuring the respiratory activity (RA) of multiple epidemiologically distinct *S.* Kentucky strains across 948 metabolic conditions (Table S1). Hierarchical clustering revealed distinct metabolic profiles, with Flu^R^ ST198 lineage strains exhibiting broader and more efficient metabolic capacities via utilization of various energy sources compared to the ST152 lineage strains (Figure 1 and Figure S1). Statistical analysis showed significantly higher RA (*p* < 0.01) for Flu^R^ ST198 strains in 17 out of 948 (1.79%) conditions, including six carbon sources, six nitrogen/sulfur sources, and five stress conditions (Table S1). The largest RA differences for Flu^R^ ST198 strains were observed with *myo*-inositol (RA difference: 247.9, *p*-value < 0.01) and lactulose (RA difference: 152.9, *p*-value < 0.01) as sole energy sources (Figure 1). In addition, Flu^R^ ST198 strains also displayed significantly higher RA (RA difference > 50, *p*-value < 0.01) when utilizing glycine, mono-methyl succinate, dextrin, and laminarin as the sole energy sources. Conversely, ST152 strains exhibited significantly higher RA with melibionic acid as the sole energy source (RA difference: 213.6, *p*-value < 0.01) (Figure 1). For nitrogen/sulfur metabolism, Flu^R^ ST198 strains outperformed ST152 strains in utilizing S-Methyl-L-Cysteine, His-Trp, Phe-Pro, Val-Ser, Gly-Gly-Gly, and His-His as sole energy sources, with no nitrogen/sulfur substrates favoring ST152 (Figure 2A). Flu^R^ ST198 strains also demonstrated great resilience under environmental stress, with higher RA under five of the six stress conditions tested, notably 4% sodium formate, pH 4.5 + L-Alanine, pH 4.5 + L-Asparagine, and pH 4.5 with or without L-Proline (Figure 2B). Conversely, ST152 strains exhibited significantly higher RA when grown with 6% NaCl with L-Carnitine (Figure 2B).

These data suggest that globally emerging Flu^R^ ST198 lineage exhibits enhanced nutritional fitness, utilizing diverse carbon sources that likely contribute to its ecological success and pathogenic potential. Similar lineage-specific metabolic divergence has been reported in other NTS serovars, such as *S.* Typhimurium ST213/ST313 and *S.* Enteritidis ST11, where lineage-specific genome degradation has been linked to distinct metabolic capacities, host adaptation, and pathogenicity [17,18,19,35,36]. For instance, the ST313 lineage of *S.* Typhimurium exhibits distinct metabolic profiles, particularly the enhanced ability to utilize several carbon compounds as sole energy sources, which likely facilitate its adaptation to host-associated niches and contribute to the ability to cause extraintestinal invasive disease [17]. Therefore, it is likely that the metabolic differences observed between ST152 and Flu^R^ ST198 reflect lineage-specific adaptations that modulate persistence in food-production systems and host environments. Overall, these results indicate that the Flu^R^ ST198 lineage possesses an expanded and more efficient capacity to utilize a range of carbon and nitrogen/sulfur substrates and enhanced tolerance and survival under a range of stressful environmental conditions, which may confer a metabolic fitness advantage in diverse or host-associated environments.

### 3.2. Lineage- and Temperature-Dependent Differences in Myo-Inositol Utilization by ST152 and Flu^R^ ST198 Strains

**Figure 3:**
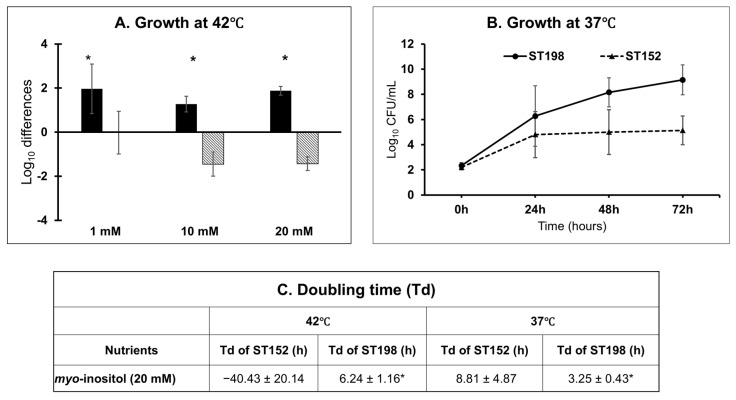
The log_10_ difference in growth of Flu^R^ ST198 (black bars) strains was significantly higher than ST152 (hatched bar) using *myo*-inositol as the sole energy source at three different concentrations at 42 °C (**A**) and using a 20 mM concentration at 37 °C (**B**). The doubling time of Flu^R^ ST198 was also significantly lower (*p* < 0.05) than ST152 at both 42 °C and 37 °C (**C**). * Indicates significant difference between Flu^R^ ST198 and ST152 under same nutrient and temperature (*p* < 0.05).

To validate the differential utilization of *myo*-inositol, growth kinetics assays were performed in media supplemented with varying concentrations (1 mM, 10 mM, and 20 mM) of *myo*-inositol at avian physiological temperature (42 °C). Flu^R^ ST198 strains exhibited significantly increased growth (1.96 ± 1.12 to 1.88 ± 0.2 log_10_ CFU/mL increase) across all concentrations of *myo*-inositol tested, with most consistent growth by all strains achieved at 20 mM concentration (Figure 3A,C). In contrast, the ST152 strains showed a consistent decline in viable counts across all concentrations at 42 °C, indicating an inability to effectively utilize *myo*-inositol as a sole energy source under avian physiological conditions.

To further evaluate temperature-dependent effects, growth assays were repeated at the mammalian physiological temperature (37 °C) using 20 mM *myo*-inositol, the concentration that supported maximal ST198 growth and has been used in previous in vitro and infection studies [37,38]. At 37 °C, Flu^R^ ST198 strains reached significantly higher cell densities (9.15 ± 1.19 log_10_ CFU/mL 72 h post-incubation with a Td of 3.25 ± 0.43 h) compared to ST152 strains (5.14 ± 1.14 log_10_ CFU/mL 72 h post-incubation with a Td of 8.81 ± 4.87 h) (Figure 3B,C). These results suggest that ST152 strains possess a markedly reduced capacity to metabolize *myo*-inositol, although they are capable of residual growth at 37 °C. The absence of growth at 42 °C but limited growth at 37 °C in ST152 strains may reflect temperature-sensitive regulation or partial functionality of pathways involved in *myo*-inositol utilization. Together, these findings indicate that Flu^R^ ST198 strains can efficiently use *myo*-inositol at both avian and mammalian physiological temperatures, whereas ST152 strains exhibit impaired and temperature-dependent utilization.

### 3.3. Lineage- and Temperature-Dependent Differences in Lactulose Utilization by ST152 and Flu^R^ ST198 Strains

**Figure 4:**
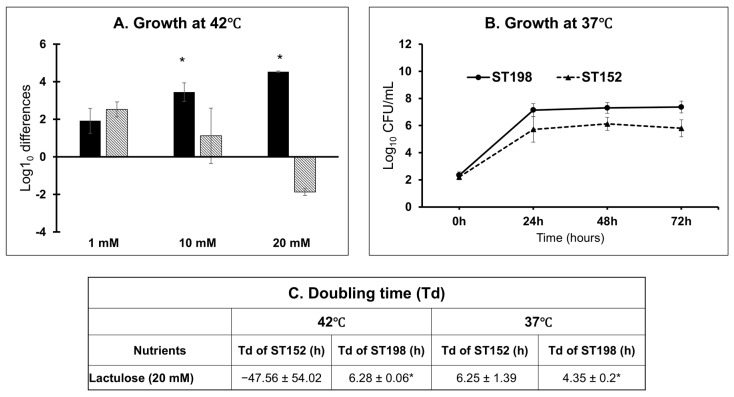
The log_10_ CFU/mL differences in growth of Flu^R^ ST198 (black bars) strains were significantly higher than ST152 (hatched bar) under lactulose as the sole energy source at 10 mM and 20 mM concentrations at 42 °C (**A**) and at 20 mM concentration at 37 °C incubation temperature (**B**). The doubling time of Flu^R^ ST198 was also significantly lower (*p* < 0.05) than ST152 at both 42 °C and 37 °C (**C**). * Indicates significant difference between Flu^R^ ST198 and ST152 under same nutrient and temperature (*p* < 0.05).

Growth kinetics assays in media supplemented with lactulose (1 mM, 10 mM, and 20 mM) at the avian physiological temperature (42 °C) revealed striking lineage-specific differences in lactulose utilization as the sole source of energy for growth. Flu^R^ ST198 strains exhibited a clear dose-dependent increase in growth, with viable counts rising from 1.92 ± 0.66 log_10_ CFU/mL at 1 mM to 3.45 ± 0.49 log_10_ CFU/mL at 10 mM and 4.54 ± 0.03 log_10_ CFU/mL at 20 mM, indicating efficient metabolism of lactulose as a sole energy source (Figure 4A). In contrast, ST152 strains showed a consistent decline in growth, from 2.53 ± 0.40 log_10_ CFU/mL at 1 mM to 1.12 ± 1.47 log_10_ CFU/mL at 10 mM, and a net loss of viability (−1.86 ± 0.20 log_10_ CFU/mL) at 20 mM, suggesting either an inability to catabolize lactulose or potential toxic effects at higher concentrations at the avian physiological temperature.

Follow-up assays at mammalian physiological temperature (37 °C) using 20 mM lactulose demonstrated that strains within both lineages were capable of growth. However, ST152 strains exhibited reduced proliferation compared to ST198 (*p* < 0.05), reaching 5.80 ± 0.63 log_10_ CFU/mL at 72 h with a doubling time of 6.25 ± 1.39 h, while Flu^R^ ST198 strains reached 7.36 ± 0.43 log_10_ CFU/mL with a faster doubling time of 4.35 ± 0.2 h (Figure 4B,C).

Taken together, these results highlight the superior metabolic flexibility of ST198, which can efficiently utilize lactulose across both avian and mammalian physiological temperatures. In contrast, ST152 shows temperature-dependent impairment in lactulose utilization with significantly reduced growth at avian physiological temperature; however, this effect is minimized at mammalian physiological temperature. These metabolic constraints suggest lactulose may differentially modulate ecological adaptation in different hosts, impacting fitness, environmental persistence, or zoonotic potential.

### 3.4. Pan-Genome and Comparative Gene Functional Analysis of ST152 and Flu^R^ ST198 Strains

**Figure 5:**
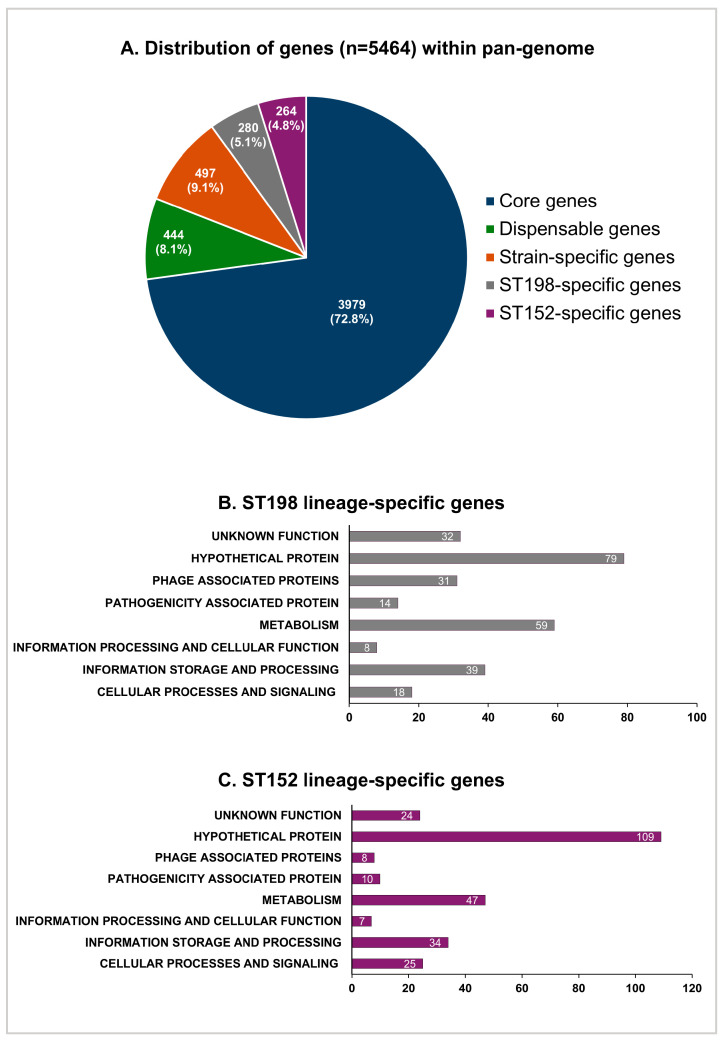
Distribution of genes (*n* = 5464) (**A**) within the pan-genome of epidemiologically distinct Flu^R^ ST198 (*n* = 3) (**B**) and ST152 (*n* = 4) strains (**C**).

The comparative genomic analysis of four strains of the ST152 lineage and three strains of the Flu^R^ ST198 lineage used in this study revealed a pan-genome consisting of 5464 genes (Figure 5A). A total of 280 (5.1%) genes were identified as ST198-lineage specific, whereas 264 (4.8%) genes were identified as ST152-lineage specific (Figure 5A). The functional distribution of ST152 and Flu^R^ ST198 lineage-specific genes revealed that each lineage carried a unique repertoire of genes, with the majority of genes encoding bacterial metabolism, hypothetical proteins, and proteins of unknown functions (Figure 5B,C). The differential carriage of the broad repertoire of genes involved in metabolic functions corroborates the differences in the metabolic phenotypes of these two lineages observed in this study. The results of the pan-genome analysis suggest that lineage-specific mutation and genome degradation may directly impact the metabolic adaptation of the sequence types in different niches, including the host and the environment. Our follow-up investigations focused on determining the linkage between genotype and phenotype for the differential ability of ST152 and Flu^R^ ST198 strains to utilize *myo*-inositol and lactulose as sole carbon sources.

#### 3.4.1. Phenotype-to-Genotype Association for *Myo*-Inositol Metabolism in *S.* Kentucky ST198 and ST152 Lineages and Across NTS

**Figure 6:**
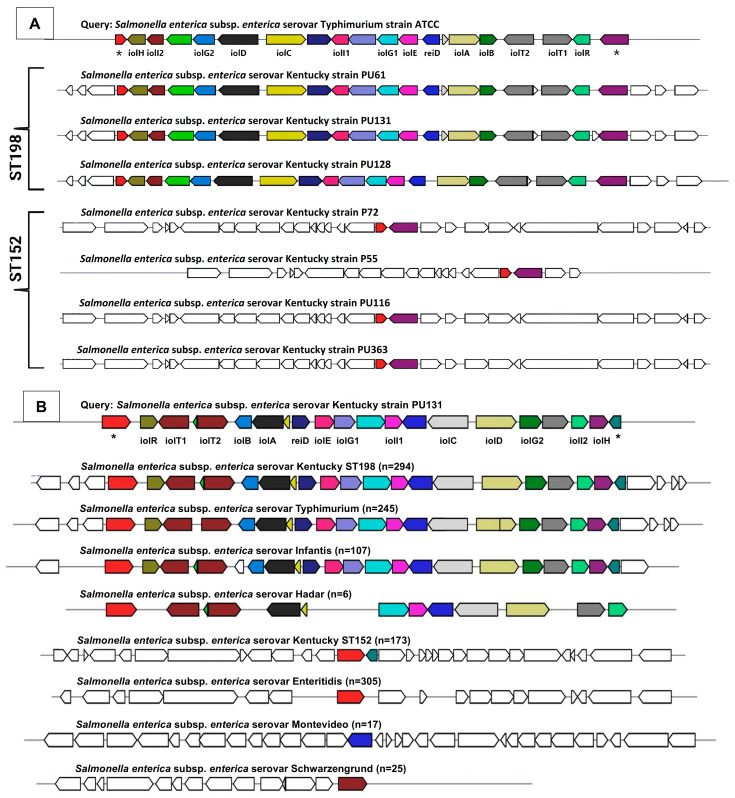
Distribution of *iolCatGC* involved in *myo*-inositol catabolism in *S.* Kentucky Flu^R^ ST198 (*n* = 3) and ST152 (*n* = 4) strains (**A**). Differential carriage of *iolCatGC* in different *Salmonella* serovars (**B**). * Flanking genes. Identical color shows identical genes.

A recent large-scale genomic survey encompassing 193,753 genomes from 24,812 commensal and pathogenic bacterial species, including *Salmonella* spp., found that *myo*-inositol utilization is widespread. This is largely attributed to the presence of a conserved *iol* gene cluster, which mediates *myo*-inositol metabolism [27,39]. Previous studies have confirmed that *S.* Typhimurium can utilize *myo*-inositol as a sole carbon source due to this canonical *iol* cluster [26,27]. In our study, strains belonging to the *S.* Kentucky ST152 lineage exhibited significantly impaired growth in *myo*-inositol minimal media compared to Flu^R^ ST198 strains, suggesting differences in the presence or functionality of the *iol* gene cluster. To investigate this genotype–phenotype association, we screened the genomes of both ST152 and Flu^R^ ST198 strains for the canonical *iol* gene cluster. All Flu^R^ ST198 genomes analyzed carried the complete canonical *iol* gene cluster (Figure 6A), consistent with their robust growth in *myo*-inositol (Figure 3). In contrast, none of the ST152 strains carried the cluster, correlating with their impaired growth (Figure 3).

**Figure 7:**
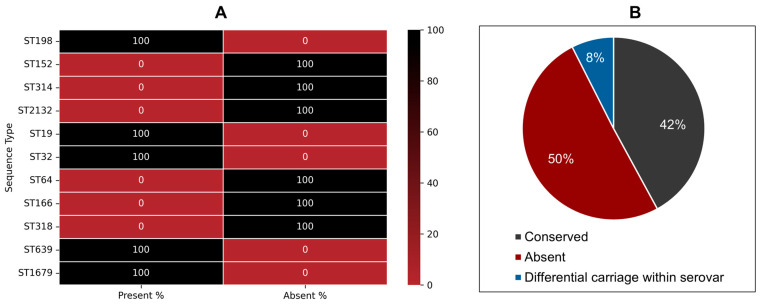
Differential carriage of inositol catabolic gene cluster *iolCatGC* across *S.* Kentucky ST198 (*n* = 294), ST152 (*n* = 173), ST314 (*n* = 14), ST2132 (*n* = 3), ST19 (*n* = 2), ST32, ST64, ST166, ST318, ST639, ST1679 (*n* = 1 each) (**A**), and 107 different NTS serovars including *S.* Kentucky (**B**).

**Figure 8:**
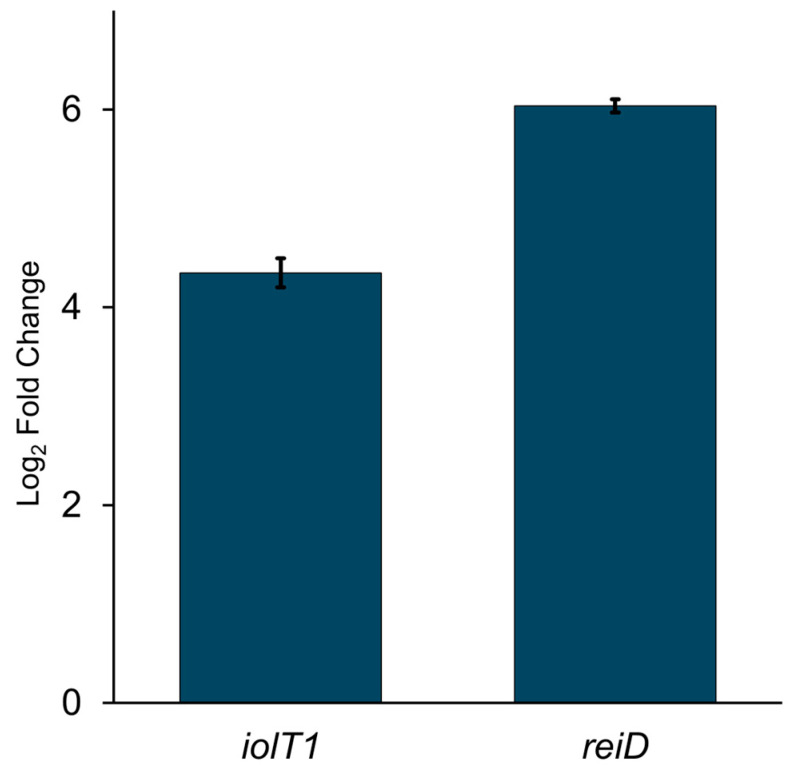
The expression of inositol catabolic genes (*iolT1* and *reiD*) was upregulated in response to *myo*-inositol when compared to glucose as a control condition.

We extended this analysis to 294 ST198 and 173 ST152 genomes to assess lineage-wide conservation. The canonical *iol* gene cluster was conserved in all ST198 genomes but absent from all ST152 genomes (Figure 7A). Other *S.* Kentucky sequence types (ST314, ST2132, ST64, ST166, and ST318) also lacked this cluster, whereas it was present in ST198, ST19, ST32, ST639, and ST1679 strains (Figure 7A). To further support this functional link, we examined the expression of two key *myo-inositol-responsive* genes [40,41], *iolT1* (transport) and *reiD* (regulatory), in the presence of *myo*-inositol. Both genes were upregulated compared to glucose conditions (Figure 8), aligning with their location in the *iol* cluster of the Flu^R^ ST198 genome (PU61) and the observed growth phenotype (Figure 3). These findings demonstrate that the canonical *iol* gene cluster is differentially distributed among *S.* Kentucky lineages and that its presence strongly correlates with the ability to utilize *myo*-inositol as a sole energy source.

**Figure 9:**
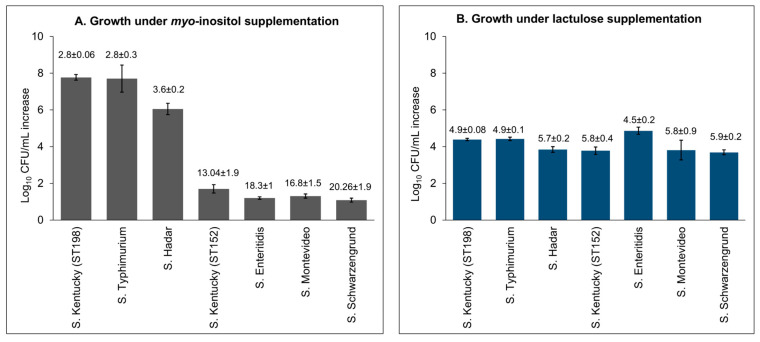
*S.* Kentucky ST198, *S.* Typhimurium, and *S.* Hadar exhibited significantly higher growth with lower Td under *myo*-inositol (20 mM) supplementation, whereas *S.* Kentucky ST152, *S.* Enteritidis, *S.* Montevideo, and *S.* Schwarzengrund had impaired growth, exhibited by lower log_10_ change with higher Td under the same conditions (**A**). All of these strains demonstrated serovar-independent growth when lactulose (20 mM) was supplemented as the sole energy source (**B**). The value above each column represents doubling time in hours at 72 h post-incubation.

To evaluate the broader relevance of this observation across NTS serovars, we analyzed 1792 genomes from 107 NTS serovars (Table S2). The canonical *iol* gene cluster was conserved in 45 (42%) serovars, absent in 54 (50%)—including *S.* Enteritidis, a clinically significant serovar, and variably present within 8 (8%) other serovars (Figure 7B). To validate these genomic predictions, representative strains were tested for growth in *myo*-inositol media. Serovars carrying the *iol* cluster, including *S.* Kentucky ST198 (7.8 ± 0.16 logs), *S.* Typhimurium (7.7 ± 0.73 logs), and *S.* Hadar (6.04 ± 0.31 logs), showed significantly higher growth than those lacking the cluster: *S.* Kentucky ST152 (1.7 ± 0.23 logs), *S.* Enteritidis (1.2 ± 0.07 logs), *S.* Montevideo (1.30 ± 0.11 logs), and *S.* Schwarzengrund (1.08 ± 0.11 logs) (Figure 9A). These findings affirm that *myo*-inositol metabolism in NTS is primarily dependent on the presence of the canonical *iol* gene cluster. Interestingly, strains lacking the cluster still demonstrated residual growth, albeit inefficiently, suggesting the potential presence of non-canonical or alternative metabolic routes for *myo*-inositol utilization. However, the molecular basis of these alternative pathways remains to be elucidated.

*Myo*-inositol, a polyol found in various foods, plays vital roles in cellular processes, including signaling, motility, membrane trafficking, and phagocytosis [27,42]. *Myo*-inositol is commonly used as a dietary supplement and for the treatment and management of several clinical conditions [43,44], and its intake ranges from 0.3 to 2.6 g/day in Western diets and up to 4.5 g/day globally [45,46], with therapeutic doses reaching 4–18 g/day [47,48]. Although typically associated with beneficial gut microbiota, recent evidence shows that several pathogenic bacterial genomes, including NTS, harbor the *iol* cluster [39]. Interestingly, *S.* Dublin has been shown to secrete a *myo*-inositol polyphosphate 4-phosphatase that disrupts host inositol phosphate signaling, contributing to diarrhea within 30 min of infection [49,50]. Our findings, showing widespread *iol* cluster carriage and associated metabolic capacity, suggest that *myo*-inositol utilization may confer a niche-specific metabolic fitness advantage to ST198 and other NTS serovars that harbor the *iol* gene cluster. Future in vivo studies are warranted to assess the contribution of this pathway to enteric colonization and pathogenesis.

#### 3.4.2. Phenotype-to-Genotype Association for Lactulose Metabolism in *S.* Kentucky ST198 and ST152 Lineages and Across NTS

While lactulose metabolism by NTS has not been previously described, our data demonstrate that NTS, including *S.* Kentucky, can utilize lactulose as a sole carbon and energy source to support growth, despite lineage-specific variation in growth efficiency (Figure 4 and Figure 9B). Growth assays confirmed that this capacity is not restricted to a single serovar; all five NTS serovars tested exhibited robust growth in media containing lactulose as the only carbon source (Figure 9B). These findings suggest that *S*. Kentucky and other NTS serovars possess a functional, yet uncharacterized, metabolic pathway for lactulose utilization.

The specific genes responsible for lactulose metabolism in NTS remain unidentified; however, glycoside hydrolase (GH) enzymes, which cleave disaccharides like lactulose into fermentable monosaccharides such as fructose and galactose, are widespread in gut microbes [51]. To identify candidate genes potentially involved in lactulose hydrolysis, we interrogated the genome of *S*. Kentucky strain PU131, a well-characterized Flu^R^ ST198 reference strain [33]. We identified six genes encoding putative GH family proteins (Table S3) and screened for their potential functions within the CAZy database. Notably, one gene (AUW48348.1) encoding a GH1 family protein with putative β-galactosidase activity was identified with the potential to hydrolyze β-1,4 linkages within disaccharides [30,31,34]. Additionally, we identified a gene (AUW48392.1) encoding a glycoside-pentoside-hexuronide (GPH) family transporter, a membrane protein family known to mediate disaccharide uptake.

**Figure 10:**
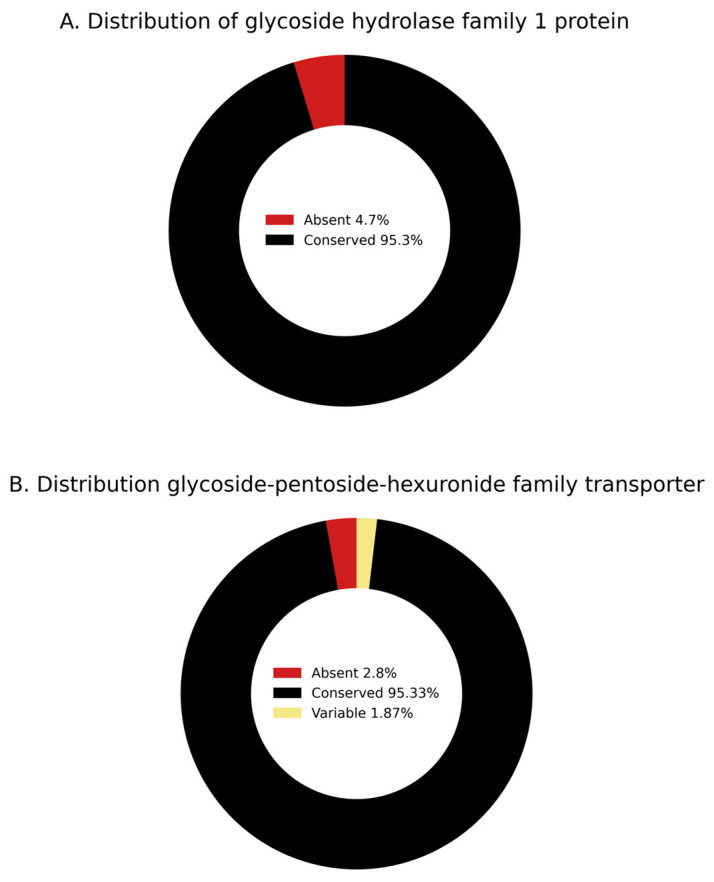
Differential carriage of Glycoside Hydrolase Family 1 (GH1) Protein (**A**) and Glycoside-pentoside-hexuronide (GPH) Family Transporter (**B**) across 107 different *Salmonella* serovars.

Comparative genomics revealed that genes encoding the GH1 enzyme and the GPH transporter are widely conserved among *S*. Kentucky isolates, including ST198 (*n* = 294), ST152 (*n* = 173), and other sequence types such as ST314 (*n* = 14) and various minor STs (*n* = 11) (Table S3). To assess broader conservation across NTS, we screened 1792 genomes representing 107 NTS serovars. The GH1 gene was present in 102 (95.3%) serovars and absent in 5 (4.7%), while the GPH transporter was also conserved in 102 (95.3%) serovars, absent in 3 (2.8%), and variably present in 2 (1.9%) (Figure 10). These results indicate that genes encoding both GH1 and GPH are broadly conserved across NTS.

The roles of these proteins in lactulose hydrolysis or uptake remain unknown. These genes are likely transcriptionally responsive to lactulose or their activity may be regulated through post-transcriptional or post-translational mechanisms [52,53]. Additionally, the CAZy database highlights that GH enzymes display high substrate specificity and are not universally present across all microbes [51,54]. Thus, it is also likely that other glycoside hydroxylase enzymes may also contribute to lactulose hydrolysis in NTS. Collectively, these data support the hypothesis that NTS may rely on a distinct and currently uncharacterized lactulose metabolic pathway. Elucidating the genetic basis of lactulose metabolism in NTS will provide key insights into the metabolic adaptability of NTS.

Lactulose is a synthetic disaccharide frequently used in clinical settings to manage hepatic encephalopathy and constipation. It is also present in heat-treated dairy products and commonly consumed as a dietary supplement at doses ranging from 10 to 60 g per day [55]. Historically, lactulose has been regarded as a prebiotic that is selectively fermented by beneficial gut bacteria, such as *Lactobacillus* and *Bifidobacterium*. However, several opportunistic pathogens, including *Escherichia*, *Enterococcus*, *Klebsiella*, *Pseudomonas*, *Streptococcus*, and *Cronobacter* species, have also been reported to metabolize lactulose [32]. Recent studies demonstrate that lactulose can enhance *Escherichia coli* (*E. coli*) colonization in both murine models and human subjects, raising concerns about a potential increased risk of systemic infection [56]. Similarly, lactulose supplementation has been shown to promote intestinal translocation of *Salmonella enterica* serovar Typhimurium [57]. In contrast, some human studies reported no significant effect of lactulose ingestion on *E. coli* abundance in stool samples of healthy volunteers [58], while others observed a reduction in potentially pathogenic *Clostridium* spp. following lactulose supplementation [59,60]. In animal models, lactulose reduced intestinal colonization by *Salmonella* and *E. coli* in chickens [61], but had no significant effect on *Salmonella* colonization in pigs [62]. Taken together, these findings suggest that the impact of lactulose on the metabolic fitness and colonization potential of enteric pathogens could be host-dependent and remains inconsistent across studies.

Our findings demonstrate that *S*. Kentucky and other NTS serovars can metabolize lactulose (Figure 4 and Figure 9B). Given the widespread use of lactulose as a prebiotic in human and veterinary contexts, these results raise important questions regarding the potential for lactulose to modulate the metabolic fitness and colonization potential of pathogenic NTS in vivo. Further studies are warranted to identify genetic determinants of lactulose metabolism in NTS and assess the mechanisms underlying lactulose-mediated modulation of NTS metabolome and its impact on NTS colonization and pathogenesis in vivo.

## 4. Conclusions

*S.* Kentucky lineages ST152 and Flu^R^ ST198 have diverged both genotypically and phenotypically, following distinct evolutionary and epidemiological trajectories. This study provides the first direct evidence of lineage-specific metabolic specialization, revealing that ST198 exhibits broader physiological plasticity, greater metabolic versatility, and enhanced stress resilience compared to ST152. These traits are evident in their efficient utilization of substrates such as *myo*-inositol and lactulose at both avian and mammalian physiological temperatures.

Our data show that ST152 lacks the canonical *iol* gene cluster responsible for *myo*-inositol catabolism in enteric bacteria, as do several other NTS serovars. Nonetheless, ST152 exhibits weak but detectable growth on *myo*-inositol, suggesting the existence of a non-canonical or alternative metabolic pathway for inositol utilization in these strains. This finding challenges the current understanding of inositol metabolism in *Salmonella* and opens new avenues for discovering novel metabolic genes or regulatory networks.

We also report for the first time that multiple NTS serovars, including *S.* Kentucky, can metabolize lactulose, a synthetic disaccharide traditionally considered fermentable only by beneficial gut microbes. The conservation of genes encoding GH1 and GPH transporter in most NTS serovars points to the potential existence of mechanisms underlying lactulose metabolism in *Salmonella*.

Together, these findings underscore the metabolic adaptability of ST198 and reveal previously unrecognized metabolic capacity in NTS. Understanding how nutrient utilization pathways contribute to ecological fitness, host adaptation, and antimicrobial resistance in NTS, and, in particular, emerging lineages like Flu^R^ ST198, is critical for developing targeted, nutrition-based interventions. Such approaches could serve as alternatives to antibiotics for controlling MDR NTS and other enteric pathogens across human and animal health interfaces.

## Figures and Tables

**Table 1:** Wild-type *S.* Kentucky and other NTS strains used in this study.

Serovar	ST Type	Strain ID	Source	Resistance	Isolation Year	Travel History	Reference
*S.* Kentucky	ST198	PU61	Human	Flu^R^	2004	Egypt, the Middle East, India, Tanzania, Ethiopia, Ivory Coast, or Morocco	Soltys et al., 2021 [9]
*S.* Kentucky	ST198	PU128	Human	Flu^R^	2013		
*S.* Kentucky	ST198	PU131	Human	Flu^R^	2013		
*S.* Kentucky	ST152	P55	Poultry	Flu^S^	2012	Not available	
*S.* Kentucky	ST152	P72	Poultry	Flu^S^	2012	Not available	
*S.* Kentucky	ST152	PU116	Human	Flu^S^	2012	No travel outside North America	
*S.* Kentucky	ST152	PU363	Poultry	Flu^S^	2005	Not available	
*S.* Typhimurium	Unknown	21611	Unknown	Not tested	Unknown	Not available	Burin and Shah, 2021 [20]
*S.* Hadar	Unknown	27032	Human	Not tested	2014	Not available	
*S.* Enteritidis	Unknown	MD15	Human	Not tested	2010	Not available	
*S.* Montevideo	Unknown	DHS-R6	Unknown	Not tested	Unknown	Not available	
*S.* Schwarzengrund	Unknown	26682	Human	Not tested	2014	Not available	

## Data Availability

All data are included in the manuscript or as Supplementary Files.

## Supplementary Materials

The following supporting information can be downloaded at: https://www.mdpi.com/article/10.3390/genes16091051/s1, Figure S1. Hierarchical clustering showing distinct metabolic profiles of ST152 and Flu^R^ ST198 strains based on the differences in respiratory activity (RA) in different conditions. Table S1. Phenotype Microarray data. Table S2. Distribution of *iolCatGC* in 107 *Salmonella* genomes. Table S3. Distribution of GH1 and GPH in 107 *Salmonella* genomes. Table S4. Primers.
