# Supplementary material for: Comparative Genomics and Functional Profiling Reveal Lineage-Specific Metabolic Adaptations in Globally Emerging Fluoroquinolone-Resistant Salmonella Kentucky ST198
Source: Genes (Basel). 2025 Sep 8;16(9):1051. doi: 10.3390/genes16091051 (PMC12469290; doi:10.3390/genes16091051)

Figure S1. Hierarchical clustering showing distinct metabolic profiles of ST152 and Flu^{R} ST198 strains based on the differences in respiratory activity (RA) in different conditions.

Figure S1a. Hierarchical clustering showing distinct metabolic profiles of ST152 and Flu^{R} ST198 strains based on the differences in respiratory activity (RA) in the presence of different nitrogen compounds as sole energy sources.

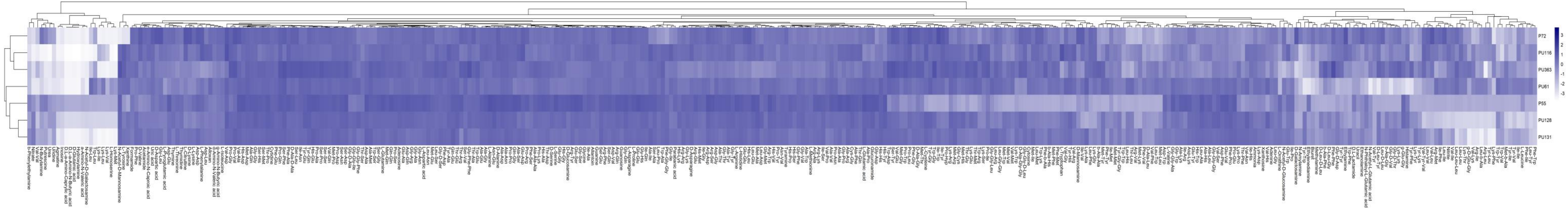

Figure S1b. Hierarchical clustering showing distinct metabolic profiles of ST152 and Flu[R] ST198 strains based on the differences in respiratory activity (RA) in the presence of different sulfur compounds as sole energy sources.

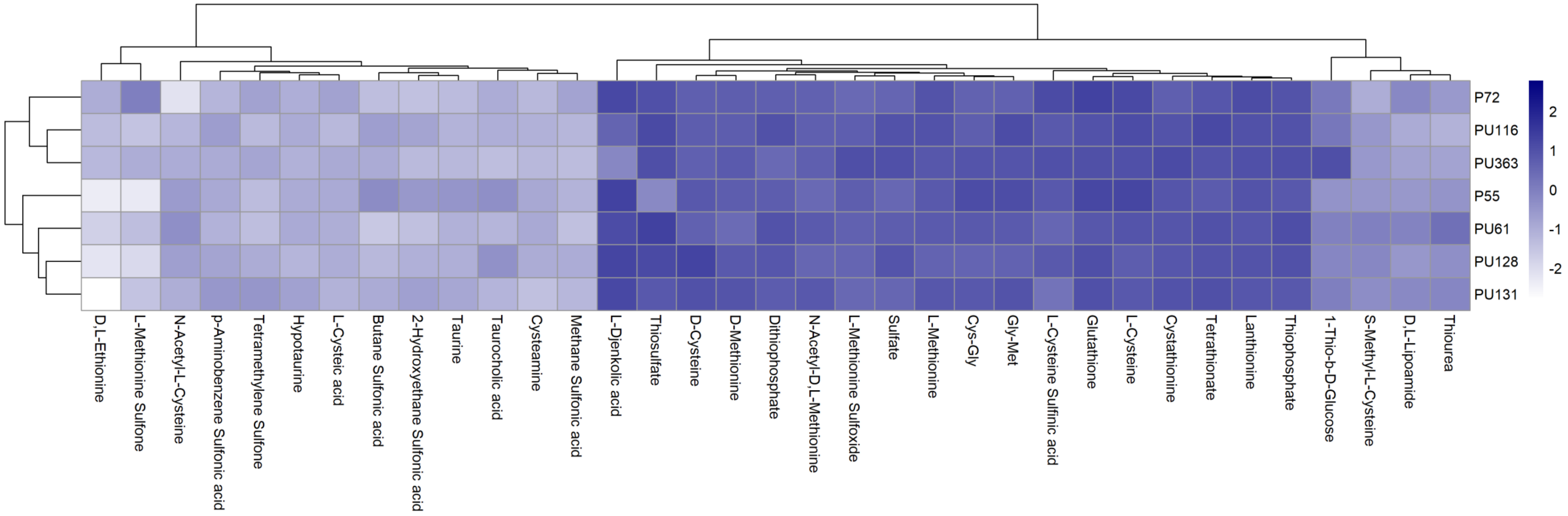

Figure S1c. Hierarchical clustering showing distinct metabolic profiles of ST152 and Flu^R ST198 strains based on the differences in respiratory activity (RA) in the presence of different phosphorus compounds as sole energy sources.

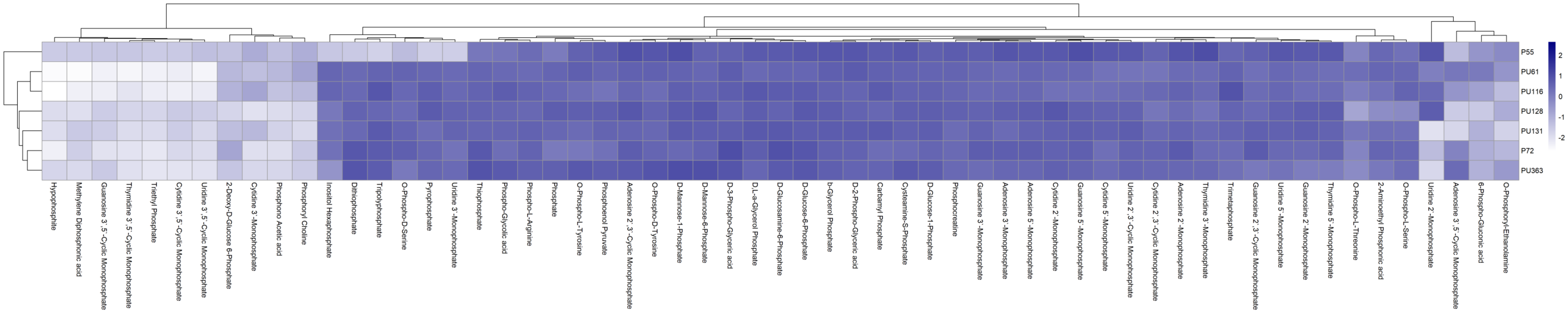

Figure S1d. Hierarchical clustering showing distinct metabolic profiles of ST152 and Flu[R] ST198 strains based on the differences in respiratory activity (RA) in the presence of different nutritional supplements as sole energy sources.

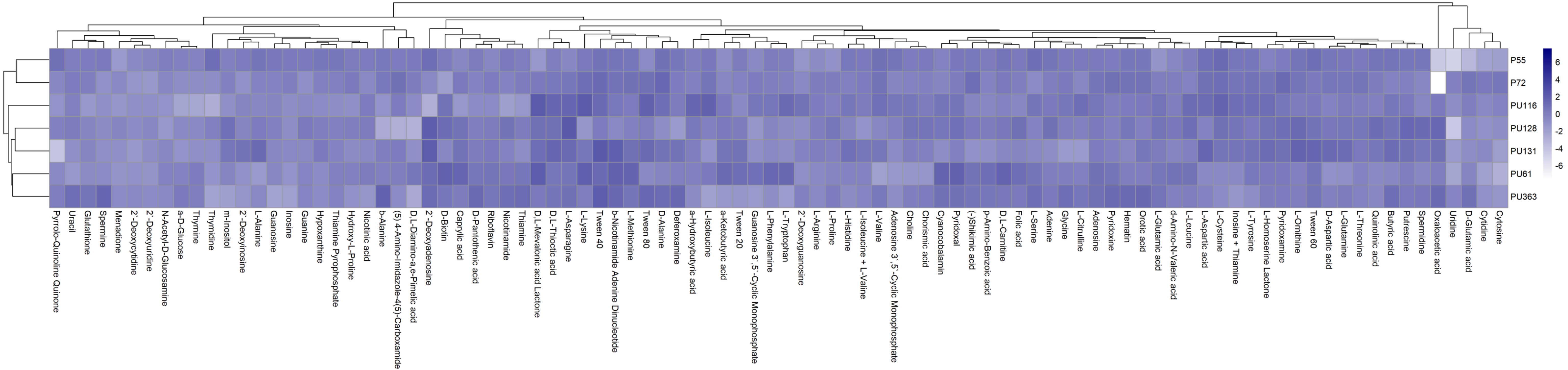

Figure S1e. Hierarchical clustering showing distinct metabolic profiles of ST152 and Flu[R] ST198 strains based on the differences in respiratory activity (RA) in the presence of different osmolytes.

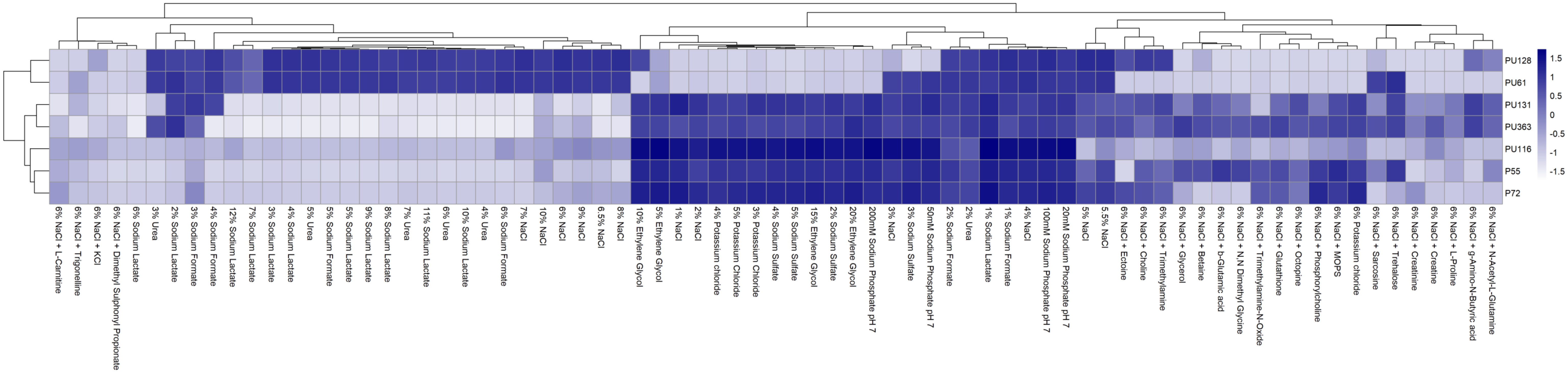

Figure S1f. Hierarchical clustering showing distinct metabolic profiles of ST152 and Flu^{R} ST198 strains based on the differences in respiratory activity (RA) in different pH conditions.

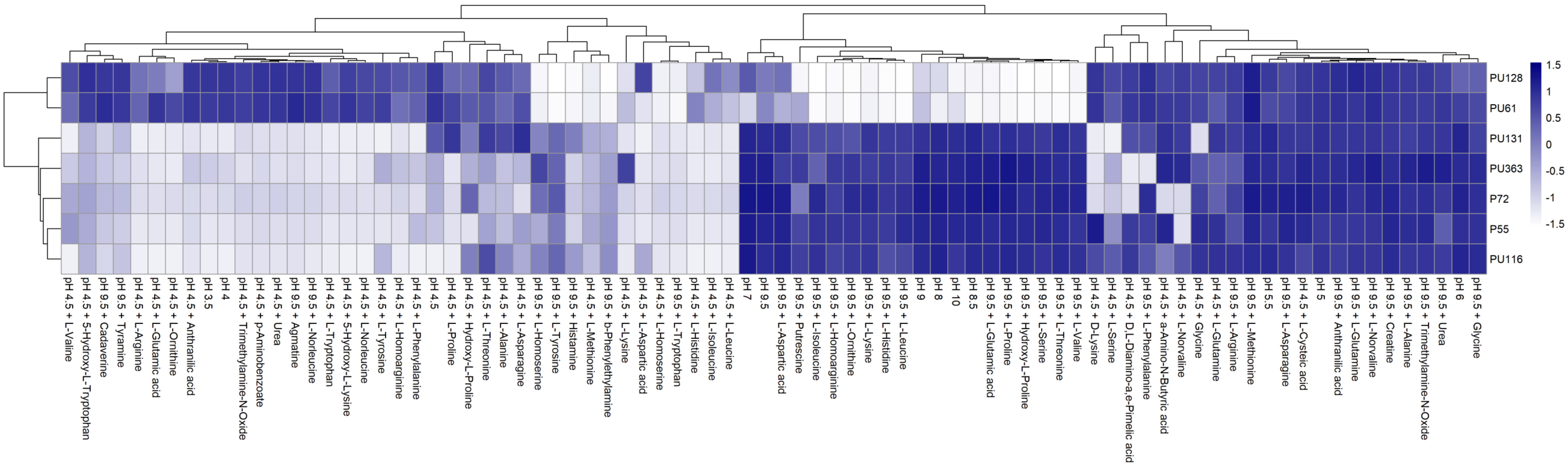

Figure S1g. Hierarchical clustering showing distinct metabolic profiles of ST152 and Flu^R ST198 strains based on the differences in respiratory activity (RA) in various toxicity.

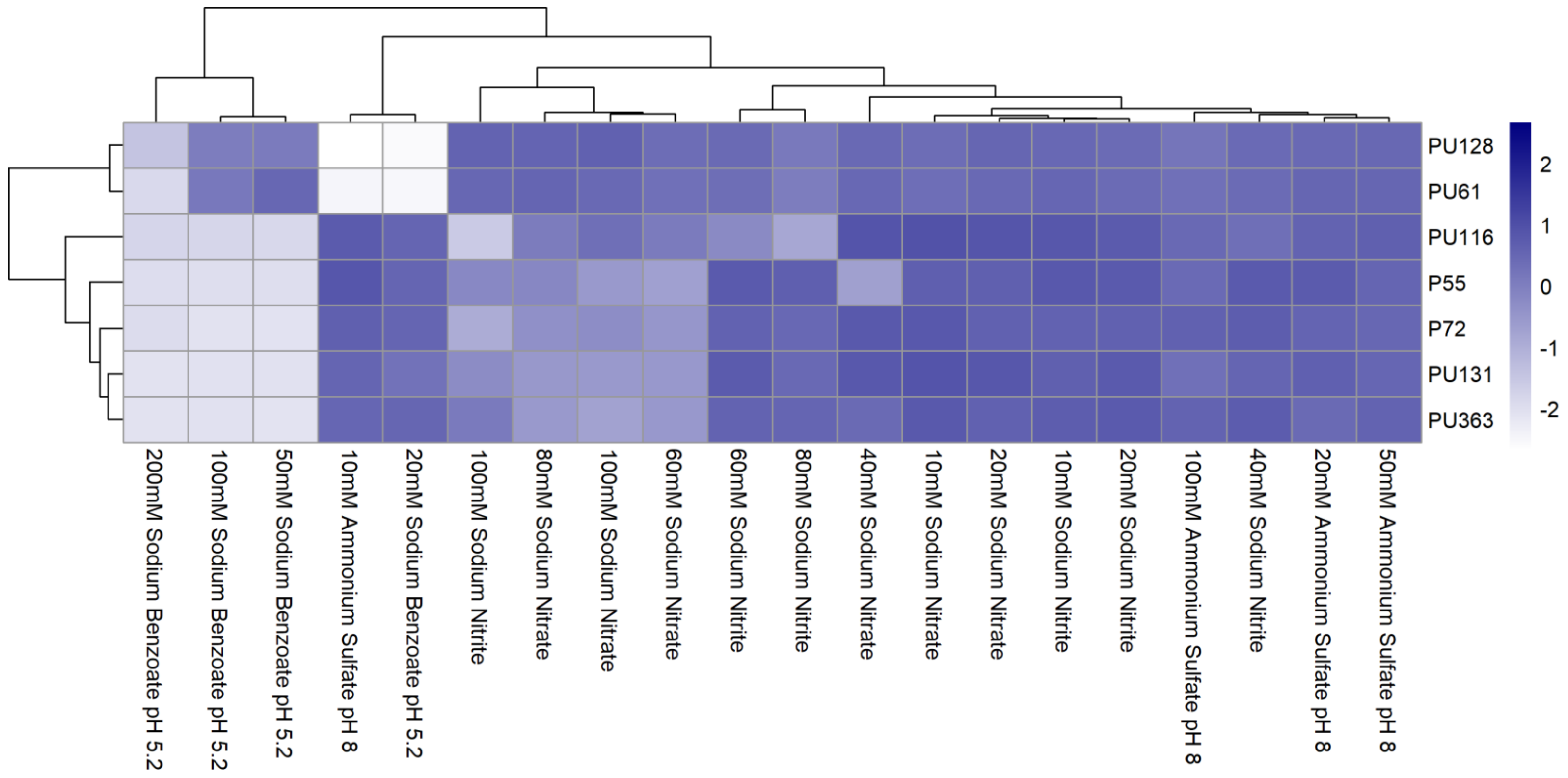

Supplement: Supplementary file 1 [file genes-16-01051-s001.zip › Figure S1.pdf]
